# The US Provided $13 Billion In Development Assistance For Health In 2016, Less Per Person Than Many Peer Nations

**DOI:** 10.1377/hlthaff.2017.1055

**Published:** 2017-12

**Authors:** Angela E. Micah, Bianca Zlavog, Sara Friedman, Alex Reynolds, Abigail L. Chapin, Matthew T. Schneider, Joseph L. Dieleman

## Abstract

Despite dramatic growth between 1990 and 2010, development assistance for health from high-income countries and development agencies to low- and middle-income countries has stagnated, and proposed cuts make future funding uncertain. To further understand international financial flows for health, we examined international contributions from major donor countries. Our findings showed that the United States provided more development assistance for health than any other country, but it provided less than others relative to national population, government spending, and income. Norway, Denmark, Luxembourg, and the United Kingdom stand out when the provision of health assistance is considered relative to these other factors. Seventeen of twenty-three countries did not reach a target that corresponds to an international goal. If all twenty-three countries had reached this goal, an additional $13.3 billion would have been available for global health in 2016. Systematic efforts are needed to encourage countries to meet these targets. Sustained health improvement in low- and middle-income countries will benefit greatly from ongoing international support.

In 2016, $37.6 billion (in 2015 US dollars) was provided by high-income countries and development agencies to maintain and improve health in low- and middle-income countries.^1^ Political support for the provision of these resources, known as development assistance for health, has faced considerable scrutiny as a result of recent political changes in major donor countries that have resurrected skepticism about bilateral and multilateral assistance.^2,3^ In the United States, the administration of President Donald Trump has proposed dramatic reductions in the foreign aid budget going forward, with a proposed cut of 24 percent for fiscal year 2018.^4,5^ There have been similar calls to reduce levels of development assistance in the United Kingdom, Denmark, the Netherlands, Norway, and other high-income nations.^6–9^ These proposals raise serious concerns about the future of international funding for global health.

Historically, there has been broad support for development assistance for health. In 2000 a global alignment in support of the Millennium Development Goals coincided with an increase in the total amount of resources targeted toward global health challenges. At its peak in 2013, the total amount of resources directed toward maintaining and improving health in low- and middle-income countries reached $38.0 billion (in 2015 US dollars), with an annualized rate of growth of 9.7 percent between 2000 and 2013. However, in recent years the annual growth rate in development assistance for health has declined, dropping 0.3 percent annually between 2013 and 2016. This slight reduction in the assistance comes despite a renewed commitment to and global alignment behind the Sustainable Development Goals—which include ambitious, expanded goals related to health to be achieved by 2030—in 2015.^10^

The provision of development assistance for health is important because in 2015 more than 87 percent of global disease burden was in low- and middle-income countries, but only 35 percent of global spending on health occurred in those countries.^11,12^ Because resources for health are so scarce in some low-income countries, development assistance for health can complement domestic funding for the provision of essential health services such as vaccinations, antiretroviral treatment, and the prevention and treatment of malaria and other diseases that affect the 6.3 billion people living in low- and middle-income countries.^13^ Such resources can be used to support the development of health system infrastructures and the training and equipping of vital health personnel.

The objective of this study was to describe the major contributors to development assistance for health, specifically highlighting how much assistance is provided by each of twenty-three countries—relative to its population, government spending, and gross national income. We examined these specific metrics because they reflect the broader country context from within which assistance is provided. Development assistance for health per person reflects the average amount of the assistance provided by the government per person in that country; the assistance per government spending indicates the share of public-sector spending allocated as such assistance; and the assistance per national income reports the share of the national economy provided as such assistance. Moreover, development assistance measured as a share of national income is the metric used by the UN General Assembly in October 1970 to set a goal for development financing flows.^14,15^

In addition to these metrics, we constructed three health-specific targets based on agreed-upon global development targets, and we report which countries are reaching each target. These analyses are important and timely because the current global health financing climate calls for an increased understanding of the context from which global health assistance has been generated.

## Study Data And Methods

**Data Sources** The primary data source used for this analysis was the development assistance for health database of the Institute for Health Metrics and Evaluation (IHME).^1,16–18^ These data track the annual disbursement of such assistance from all major international development agencies that provide it. The disbursements may be financial (in the form of grants or low-cost loans) or nonfinancial (such as the provision of technical assistance). The estimates in the database are generated using data from publicly available databases that track development assistance. These sources include project-level records, budget statements, audited financial statements, and online databases and annual reports from development agencies such as the World Bank, the Organization for Economic Cooperation and Development (OECD), and the Bill & Melinda Gates Foundation.

The IHME database disaggregates the annual disbursements of development assistance for health by the source of the funds, disbursing agency (also known as the channel), targeted health focus area, and recipient country. The source defines the origin of the funds. Sources include treasuries of high-income countries, debt repayments for loans provided by development banks, and private donations and endowments from foundations and other philanthropic entities. Data on development assistance for health by source are available for the period 1990–2016. The disaggregation by source captures twenty-three high-income countries; the European Commission; the Bill & Melinda Gates Foundation; and entities that contribute to United Nations agencies, multilateral development agencies, private US foundations, and nongovernmental organizations (NGOs). The high-income countries included are listed in online Appendix 1.1.^19^ When disbursement data were not available, data on donor commitments and budgets were adjusted to reflect disbursements. This was the case particularly for 2015 and 2016, when many donors had not yet reported their accounting of the most recent years’ disbursements. The database on development assistance for health tracks resources from source to disbursing agency and then to recipient agency to avoid double-counting associated with development agencies’ transferring resources among themselves.^16^

In addition to the data, we extracted gross national income and all-sector development assistance estimates from the OECD’s online database.^20,21^ All-sector government expenditure data were extracted from IHME’s Global Burden of Disease covariate database.^12,22^ For all three sets of data, estimates from 1990 through 2016 were extracted. Estimates were converted to 2015 US dollars using exchange rate data and deflator series from the International Monetary Fund.^23^

We report total development assistance for health provided by each of the twenty-three donor countries included in IHME’s development assistance for health database. In addition, as noted above, we calculated the assistance provided by the government of each donor country in three ways: per person, per million dollars of government spending, and per million dollars of gross national income. We also assessed the provision of assistance relative to three potential targets. All three targets were based upon the goal of contributing 0.7 percent of gross national income as official development assistance. This goal—initially included in a United Nations General Assembly resolution in 1970—has been affirmed at other international conferences, including the 2002 International Conference on Financing and Development in Monterrey, Mexico.^14,15,24^ To determine associated targets for the amount of development assistance that should be provided specifically for health, we assessed the share of official development assistance each country provided for health in 2016. We calculated the twenty-fifth, fiftieth, and seventy-fifth percentiles of this share across the twenty-three countries and multiplied these values by 0.7 percent to estimate the share of gross national income that countries should target specifically for health in low- and middle-income countries.

Few countries contributed at levels that are in line with previously agreed-upon international targets.

**Limitations** The limitations of this study revolved around data availability and quality. First, the data set we used did not include disbursements of development assistance for health from middle-income countries. Historically, transfers of such assistance have been from high-income countries to low- and middle-income countries. For high-income donor countries, which report data publicly to the OECD, the database we used is comprehensive. However, more recently, the contributions of emerging economies such as China are growing in importance and magnitude.^25^ Nonetheless, publicly available data on China’s disbursement of development assistance for health are limited, so our current data set does not include China’s contributions.

Second, although the OECD’s Development Assistance Committee database is an authoritative source of data on official development assistance, its ability to disaggregate spending by sector and its completeness, in particular for estimates from the 1990s, are imperfect. IHME mitigated these limitations by supplementing the information in this database with data from additional sources, such as projects’ disbursement data—which were accessed from international agencies such as the Global Fund to Fight AIDS, Tuberculosis, and Malaria and the World Bank and from annual reports from the OECD’s Development Assistance Committee.

Third, because of the lag in reporting and data release times for some development agencies, IHME relied on estimation and approximation to fill in incomplete data series. These estimations were necessary only in limited cases and were based on the available historical data.

Lastly, IHME’s development assistance for health database tracked health assistance in terms of total project costs and not in terms of what was actually spent in recipient countries. To the extent that actual expenditures in recipient countries deviated from the total project costs reported, our estimates may have included some measurement error.

## Study Results

Between 1990 and 2016, development assistance for health to low- and middle-income countries increased more than fivefold, from $7.1 billion to $37.6 billion (in 2015 US dollars). Exhibit 1 shows the evolution of the assistance, disaggregating contributions by source. The top three countries providing the assistance in 2016 were the United States, the United Kingdom, and Germany, which provided $12.8, $4.1, and $1.5 billion respectively. The largest annual increases occurred between 2000 and 2010. During this period, these three sources increased their spending at an annualized rate of 16 percent, 9 percent, and 12 percent, respectively.

**EXHIBIT 1:**
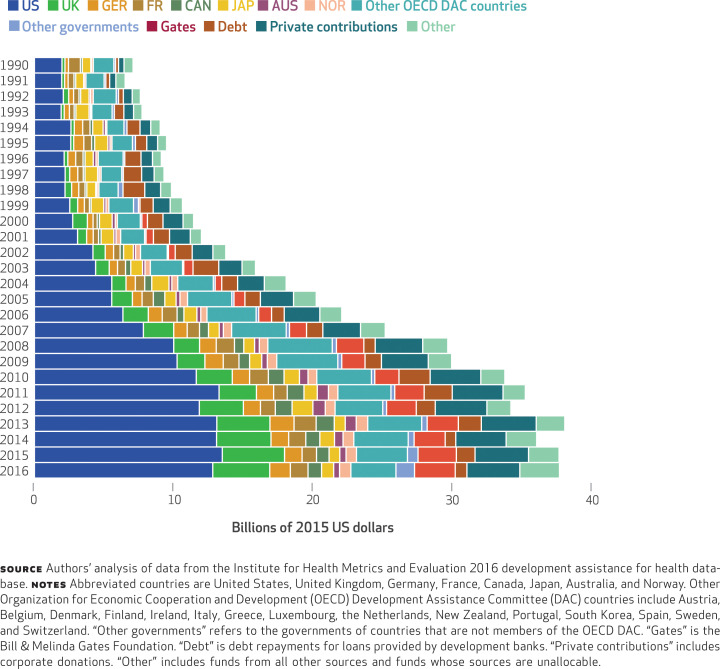
Total development assistance for health, by funding source, 1990–2016

**SOURCE** Authors’ analysis of data from the Institute for Health Metrics and Evaluation 2016 development assistance for health database. **NOTES** Abbreviated countries are United States, United Kingdom, Germany, France, Canada, Japan, Australia, and Norway. Other Organization for Economic Cooperation and Development (OECD) Development Assistance Committee (DAC) countries include Austria, Belgium, Denmark, Finland, Ireland, Italy, Greece, Luxembourg, the Netherlands, New Zealand, Portugal, South Korea, Spain, Sweden, and Switzerland. “Other governments” refers to the governments of countries that are not members of the OECD DAC. “Gates” is the Bill & Melinda Gates Foundation. “Debt” is debt repayments for loans provided by development banks. “Private contributions” includes corporate donations. “Other” includes funds from all other sources and funds whose sources are unallocable.

Appendix Exhibit A1 presents the evolution of the share of total development assistance for health contributed by the various funding sources.^19^ In 2016 the United States, the United Kingdom, and Germany provided 34 percent, 11 percent, and 4 percent, respectively, of the total assistance. While the United States has provided the largest share over the entire study period, the shares of assistance contributed by funding sources has changed. The United Kingdom’s and Canada’s contributions have progressively made up higher percentages of the total assistance contributed since 2000. Over the same period, the percentages contributed by Japan and Australia have decreased.

Exhibit 2 illustrates the channels and health focus areas that each major source provided resources for in 1990–2016. Each funding source has a distinct pattern of channels used and health focus areas supported. The United States disburses the majority of its development assistance for health through nongovernmental agencies and its bilateral agencies, including the Agency for International Development, and focuses its assistance on HIV/AIDS and maternal and child health. The United Kingdom also prioritizes its bilateral agency and NGOs, as well as agencies of the United Nations. In recent years the United Kingdom has also prioritized Gavi, the Vaccine Alliance, and the Global Fund. Japan, the third-largest source of the assistance in the whole study period, chiefly uses its own bilateral agency and prioritizes other health focus areas.

**EXHIBIT 2:**
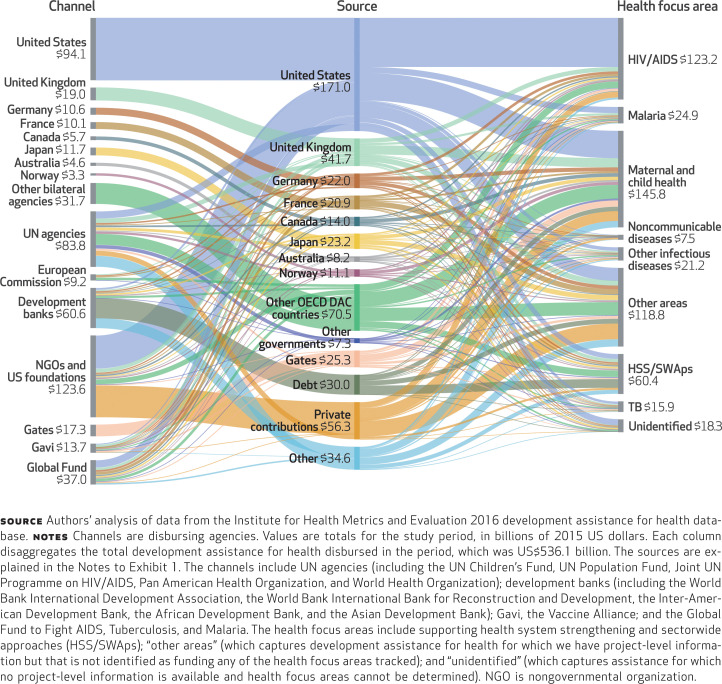
Flow of funds (billions) for development assistance for health from sources to both channels and health focus areas, 1990–2016

**SOURCE** Authors’ analysis of data from the Institute for Health Metrics and Evaluation 2016 development assistance for health database. **NOTES** Channels are disbursing agencies. Values are totals for the study period, in billions of 2015 US dollars. Each column disaggregates the total development assistance for health disbursed in the period, which was US$536.1 billion. The sources are explained in the Notes to Exhibit 1. The channels include UN agencies (including the UN Children’s Fund, UN Population Fund, Joint UN Programme on HIV/AIDS, Pan American Health Organization, and World Health Organization); development banks (including the World Bank International Development Association, the World Bank International Bank for Reconstruction and Development, the Inter-American Development Bank, the African Development Bank, and the Asian Development Bank); Gavi, the Vaccine Alliance; and the Global Fund to Fight AIDS, Tuberculosis, and Malaria. The health focus areas include supporting health system strengthening and sectorwide approaches (HSS/SWAps); “other areas” (which captures development assistance for health for which we have project-level information but that is not identified as funding any of the health focus areas tracked); and “unidentified” (which captures assistance for which no project-level information is available and health focus areas cannot be determined). NGO is nongovernmental organization.

Exhibit 3 shows total development assistance for health provided by each government, and the total amount disbursed relative to the size of the population, total government spending, and gross national income. These four metrics of development assistance for health provided by each country places the disbursement of the assistance in a different context. While the United States and the United Kingdom provided more of the assistance than any other country, other nations stood out as providing more assistance per person, per government expenditure, and per national income. Assistance contributed per person measures the actual burden of providing the assistance on citizens of the contributing country. The citizens of Norway and Luxembourg contribute over $100 per person toward the assistance, while US citizens contribute $41 per person. Norway and the United Kingdom devote the highest amounts toward global health per $1 million of general government spending, dedicating $4,956 and $4,677, respectively. Norway, Denmark, Luxembourg, and the United Kingdom are the most generous providers of development assistance for health: Each of these countries contributes more than $1,500 per $1 million in national income. By this metric, the United States is the seventh most generous contributor.

**EXHIBIT 3:**
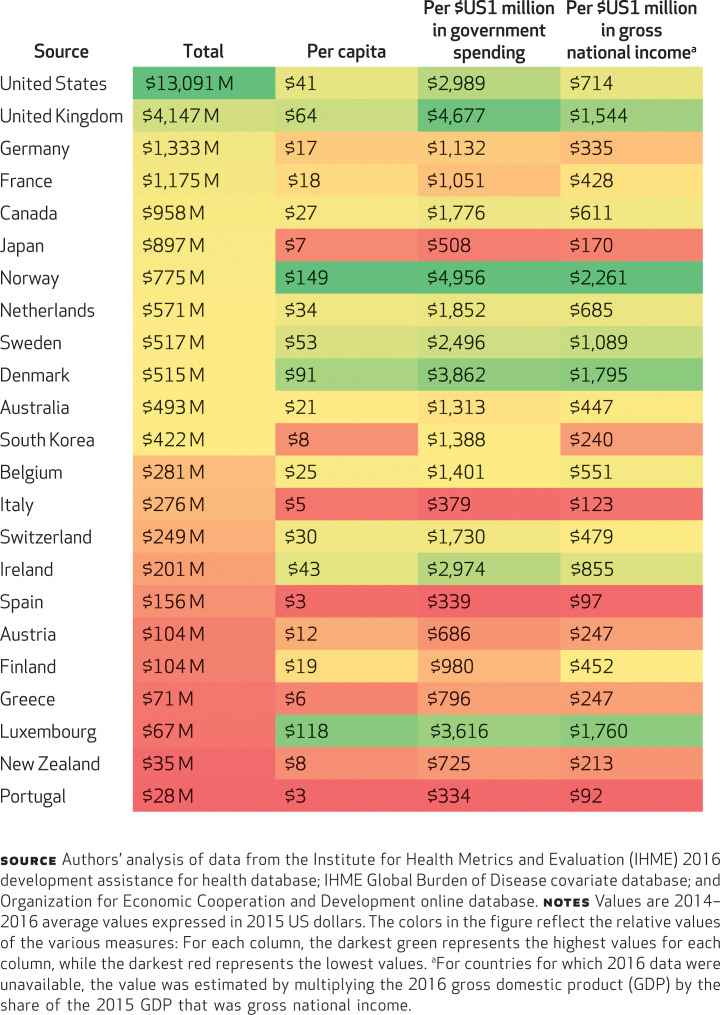
Four measures of development assistance for health, 2014–2016

**SOURCE** Authors’ analysis of data from the Institute for Health Metrics and Evaluation (IHME) 2016 development assistance for health database; IHME Global Burden of Disease covariate database; and Organization for Economic Cooperation and Development online database. **NOTES** Values are 2014–2016 average values expressed in 2015 US dollars. The colors in the figure reflect the relative values of the various measures: For each column, the darkest green represents the highest values for each column, while the darkest red represents the lowest values. ^a^For countries for which 2016 data were unavailable, the value was estimated by multiplying the 2016 gross domestic product (GDP) by the share of the 2015 GDP that was gross national income.

Overall, development assistance for health is very small when compared to total domestic health spending in donor countries. In Norway, where the highest relative percentage was observed, international health spending was only 2.3 percent of what the country spent on domestic health (Appendix Exhibit A2).^19^ In the United States, development assistance for health accounted for only 0.4 percent.

Exhibit 4 shows the share of national income that goes toward global health assistance for 2014–16. Nine of the twenty-three countries did not meet the lowest health-specific target level of spending. Seven additional countries surpassed the lowest target, but only six countries met the medium target. If the remaining seventeen countries had met that relatively modest target, an additional $13.3 billion could have been available for global health each year in 2014–16.

**EXHIBIT 4:**
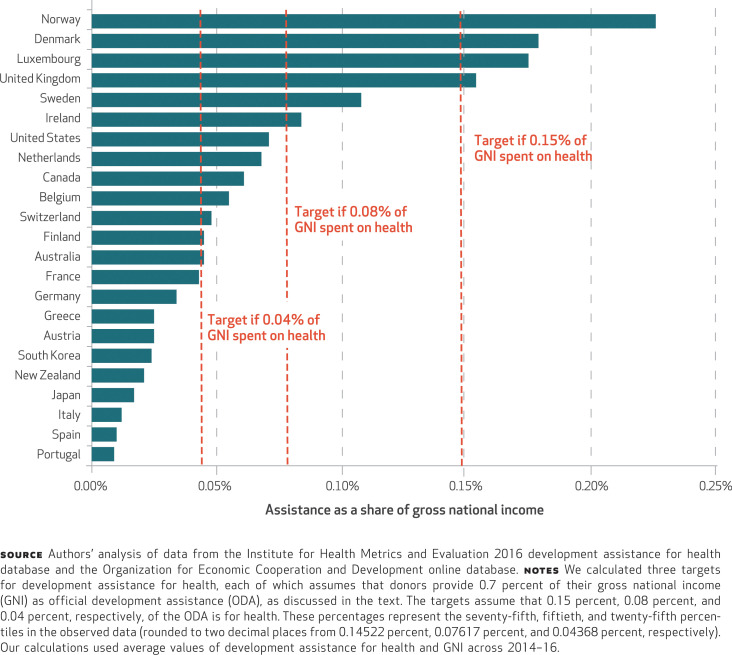
Development assistance for health relative to national income and health-specific targets, 2014–2016

**SOURCE** Authors’ analysis of data from the Institute for Health Metrics and Evaluation 2016 development assistance for health database and the Organization for Economic Cooperation and Development online database. **NOTES** We calculated three targets for development assistance for health, each of which assumes that donors provide 0.7 percent of their gross national income (GNI) as official development assistance (ODA), as discussed in the text. The targets assume that 0.15 percent, 0.08 percent, and 0.04 percent, respectively, of the ODA is for health. These percentages represent the seventy-fifth, fiftieth, and twenty-fifth percentiles in the observed data (rounded to two decimal places from 0.14522 percent, 0.07617 percent, and 0.04368 percent, respectively). Our calculations used average values of development assistance for health and GNI across 2014–16.

## Discussion

This study identified the nations that collectively provide a majority of development assistance for health, using data for the period 1990–2016. In particular, it examined how much of the assistance was provided by twenty-three nations, as well as how much of it was provided relative to the size of each country’s population, government spending, and economy. The United States provided more assistance than any other country, but relative to the size of the population, government spending, and national income, Norway, Denmark, Luxembourg, and the United Kingdom stand out as leaders. Each of the leading sources of assistance focused resources through its bilateral aid agency, although each also supported a diversity of multilateral agencies, UN organizations, public-private partnerships, and NGOs. These leaders also allocated assistance to a diverse group of health focus areas, with some countries, such as the United States, focusing assistance on a few primary health areas. Most concerning, few countries contributed at levels that are in line with previously agreed-upon international targets. These findings highlight the gap between these targets and the actual assistance provided that could be used to generate more resources in support of critical health services, especially in low-income countries.

Each of the four metrics used to measure the amount of development assistance for health provided by a given country places disbursement of the assistance in a different context. The total amount provided by all countries represents the amount of resources available for improving health. This highlights the amount of resources provided and which countries are supporting health care services in low- and middle-income countries the most. This research reveals that the United States and the United Kingdom provide substantially more of the assistance than any other country.

Other metrics—the assistance contributed per person, per government spending, and per national income—provide additional information about the context within which these contributions were made and can be used for comparisons and goal setting. For instance, in terms of how much assistance is provided per person, Norwegians and Luxembourgers contribute the most, each contributing nearly 50 percent more than citizens of any other countries and more than 200 percent of what each American contributes. Development assistance for health as a share of government spending highlights how much of the public budget is allocated to development assistance for health. Norway, Denmark, and the United Kingdom rank the highest. All three of these countries contribute substantially more than the United States. Finally, assistance per national income is an important measure, showing how much of the country’s income goes toward global health assistance. On this metric Norway, Denmark, and the United Kingdom again stand out—in this case, joined by Luxembourg—as providing more than double the assistance provided by the United States, with Norway providing over three times as much as the United States.

These findings show that although the United States provided more in terms of total amount, Norway, the United Kingdom, Denmark, and Luxembourg provided more of the assistance relative to population size, public spending, and size of the economy. This distinction is important because current discussions of the assistance typically center on the amount of assistance contributed, whereas contextualizing contributions presents a picture of what is realistic and feasible given each country’s position. The 2002 International Conference on Financing and Development in Monterrey at which many development partners reaffirmed the targets for official development assistance is one such attempt to resize the development assistance pie based on country context.^24^

In addition to pointing out the countries that provide the most development assistance for health, this research also highlights the global dependence on many countries.While the United States and the United Kingdom together provided 45 percent of all the assistance in 2016, many other countries and other contributors together make up the remainder of global health financing. Recent fund-raising efforts by major international development agencies such as the World Bank’s International Development Association, the Global Fund, and Gavi have highlighted contributions from many small countries.^26–29^ Such efforts emphasize the important role that all countries play in providing the assistance.

While uncertainty about the future provision of development assistance for health looms large, this research highlights the fact that most countries are not meeting already agreed-upon funding targets. Of the twenty-three countries in this analysis, seventeen—including the United States, Canada, France, Germany, and Japan—fell short of the medium target explored in this research. If these seventeen countries had met this relatively modest target, an additional $13.3 billion could have been available for global health in 2016. While these gaps may reflect the climate of increased skepticism, they also present a unique opportunity for the international community to renew its commitment to, and increase the resources that support, global health goals.

## Policy Implications

The current global health funding environment exposes the vulnerable and precarious nature of external funding for global health. A change in a country’s ability or willingness to provide these resources in the future could have significant implications for the total amount of development assistance for health. For instance, the proposed cuts in the 2018 US federal budget would have dire implications for key areas of global health where substantial progress has been made, particularly because of the large portion of the total assistance that has been provided by the US government. A recent budget impact analysis estimated that the cuts could result in 49,100–198,700 increases in new HIV infections in recipient countries by July of 2019.^4^ Similarly, there could be an increase of 7,600–31,000 new TB cases.^4^ Maternal, newborn, and child deaths would also increase.^4^ Although there has generally been a broad base of support for the provision of development assistance for health across the political spectrum in the United States and elsewhere, the recent political focus on reducing global health funding has led those who track global health financing to sound the alarm. These potential scenarios present an uncertain future for the trajectory of the assistance and the millions of people in low- and middle-income countries who rely on it to meet their essential health care needs.

Moving forward, there will also likely be more emphasis on assistance provided by middle-income countries such as Brazil, China, and Russia. Already some estimates and development institutions have suggested that China may be providing a meaningful amount of development assistance for health.^30,31^ These new sources of funds, along with major private donors such as the Bill & Melinda Gates Foundation and public-private partnerships such as the Global Fund and Gavi, will supplement resources provided by the traditional sources of development assistance for health. However, it will likely be some time before these donors provide as many resources as the United States and the United Kingdom, currently the largest donors.With this in mind, it is important that the global community encourage all members, including the United States and the United Kingdom, to provide what they can for global health, targeting aid toward the people most in need. To ensure meaningful international comparisons, we encourage ongoing benchmarking that prioritizes assessing countries’ contribution in light of their national context. Targets—such as those adopted by many at the 2002 International Conference on Financing and Development in Monterrey—that set goals relative to national income can be useful for considering a country’s provision of development assistance relative to its own economy and wealth.

## Conclusion

Recent national and international events have generated uncertainty regarding the future of global health contributions. Our analysis highlights the main contributors to development assistance for health, focusing on each country’s provision of the assistance relative to its population, government spending, and national income. This analysis is useful because it provides evidence to broaden the content and context of discussions of global health funding. The findings from the analysis suggest that although the US contribution to development assistance for health is the largest in the aggregate, that contribution in relation to the size of national population, government spending, and national economy is notably less than that of some other countries. Nonetheless, the proposed cuts by the United States, a major contributor, will have far-reaching consequences for millions of people around the world. Additionally, given that many countries, including the United States, make contributions that are below the agreed-upon targets, global efforts are needed to make progress toward reaching the targets and providing resources for addressing important global health goals.

This research was supported by the Bill & Melinda Gates Foundation. The funding organization had no role in the design and conduct of the study; collection, management, analysis, and interpretation of the data; preparation, review, or approval of the manuscript; or decision to submit the manuscript for publication. This is an open access article distributed in accordance with the terms of the Creative Commons Attribution (CC BY 4.0) license, which permits others to distribute, remix, adapt and build upon this work, for commercial use, provided the original work is properly cited. See: https://creativecommons.org/licenses/by/4.0/

## NOTES

1 Dieleman JL, Campbell M, Chapin A, Eldrenkamp E, Fan VY, Haakenstad A, et al. Evolution and patterns of global health financing 1995–2014: development assistance for health, and government, prepaid private, and out-of-pocket health spending in 184 countries. Lancet. 2017; 389(10083):1981–2004.

2 Anders M. New DfID head divides UK aid community over trade focus Devex [serial on the Internet]. 2016 Aug 4 [cited 2017 Nov 1]. Available from: https://www.devex.com/news/sponsored/new-dfid-head-divides-uk-aid-community-over-trade-focus-88531

3 Murphy T. Tillerson’s “less is more” strategy for foreign aid meets bipartisan skepticism in Senate. Humanosphere [serial on the Internet]. 2017 Jun 14 [cited 2017 Nov 1]. Available from: http://www.humanosphere.org/basics/2017/06/tillersons-less-is-more-strategy-for-foreign-aid-meets-bipartisan-skepticism-in-senate/

4 Kates J, Wexler A, Michaud J, Stover J. What could U.S. budget cuts mean for global health? [Internet]. Menlo Park (CA): Henry J. Kaiser Family Foundation; 2017 Jul [cited 2017 Nov 1]. (Issue Brief). Available from: http://files.kff.org/attachment/Issue-Brief-What-Could-US-Budget-Cuts-Mean-for-Global-Health

5 Henry J. Kaiser Family Foundation. The U.S. government and global health [Internet]. Menlo Park (CA): KFF; 2017 Aug 8 [cited 2017 Nov 1]. Available from: http://www.kff.org/global-health-policy/fact-sheet/the-u-s-government-and-global-health/

6 Foreign aid: UKIP calls for 80% cut in annual spending. BBC News [serial on the Internet]. 2017 Feb 16 [cited 2017 Nov 1]. Available from: http://www.bbc.com/news/uk-politics-38983952

9 Anders M. Norway diverting aid to cover refugees: robbing Peter to pay Paul? Devex [serial on the Internet]. 2015 Nov 6 [cited 2017 Nov 1]. Available from: https://www.devex.com/news/sponsored/norway-diverting-aid-to-cover-refugees-robbing-peter-to-pay-paul-87252

10 United Nations. Sustainable development goals [Internet]. New York (NY): UN; [cited 2017 Nov 1]. Available from: http://www.un.org/sustainabledevelopment/sustainable-development-goals/

11 GBD 2015 DALYs and HALE Collaborators. Global, regional, and national disability-adjusted life-years (DALYs) for 315 diseases and injuries and healthy life expectancy (HALE), 1990–2015: a systematic analysis for the Global Burden of Disease Study 2015. Lancet. 2016;388(10053): 1603–58.

12 GHDx. Forecasting global development assistance for health, government, prepaid private, and out-of-pocket health spending 2015–2040 [Internet]. Seattle (WA): Institute for Health Metrics and Evaluation; [last modified 2017 Sep 6; cited 2017 Nov 1]. Available from: http://ghdx.healthdata.org/record/forecasting-global-development-assistance-health-government-prepaid-private-and-out-pocket

13 World Bank. Low and middle income [Internet]. Washington (DC): World Bank; c 2017 [cited 2017 Nov 1]. Available from: http://data.worldbank.org/income-level/low-and-middle-income

14 United Nations. Resolution adopted by the General Assembly 2626 (XXV). International Development Strategy for the Second United Nations Development Decade [Internet]. New York (NY): UN; 1970 Oct 24 [cited 2017 Nov 7]. Available from: http://www.un-documents.net/a25r2626.htm

15 Organization for Economic Cooperation and Development. The 0.7% ODA/GNI target—a history [Internet]. Paris: OECD; [cited 2017 Nov 13]. Available from: http://www.oecd.org/dac/stats/the07odagnitarget-ahistory.htm

16 GHDx. Development assistance for health database 1990–2016 [Internet]. Seattle (WA): Institute for Health Metrics and Evaluation; [last modified 2017 Sep 6; cited 2017 Nov 7]. Available from: http://ghdx.healthdata.org/record/development-assistance-health-database-1990-2016

18 Dieleman JL, Campbell M, Chapin A, Eldrenkamp E, Fan VY, Haakenstad A, et al. Future and potential spending on health 2015–40: development assistance for health, and government, prepaid private, and out-of-pocket health spending in 184 countries. Lancet. 2017;389(10083): 2005–30.

19 To access the Appendix, click on the Details tab of the article online.

20 Organization for Economic Cooperation and Development. Net ODA [Internet]. Paris: OECD; c 2016 [cited 2017 Nov 1]. Available from: http://data.oecd.org/oda/net-oda.htm

21 Organization for Economic Cooperation and Development. Gross national income [Internet]. Paris: OECD; c 2016 [cited 2017 Nov 1]. Available from: http://data.oecd.org/natincome/gross-national-income.htm

22 GHDx. Global development assistance for health, government, prepaid private, and out-of-pocket health spending 1995–2014 [Internet]. Seattle (WA): Institute for Health Metrics and Evaluation; last modified 2017 Sep 6 [cited 2017 Nov 1]. Available from: http://ghdx.healthdata.org/record/global-development-assistance-health-government-prepaid-private-and-out-pocket-health

23 International Monetary Fund. World Economic Outlook database [Internet]. Washington (DC): IMF; [cited 2017 Nov 1]. Available from: https://www.imf.org/external/pubs/ft/weo/2017/01/weodata/index.aspx

24 United Nations. Monterrey consensus on financing for development [Internet]. New York (NY): UN; 2003 [cited 2017 Nov 1]. Available from: http://www.un.org/esa/ffd/monterrey/MonterreyConsensus.pdf

25 Troilo P. The building BRICs: analyzing the role of the BRIC countries in foreign aid and global development. Devex [serial on the Internet]. 2011 Oct 11 [cited 2017 Nov 1]. Available from: https://www.devex.com/news/sponsored/the-building-brics-analyzing-the-role-of-the-bric-countries-in-foreign-aid-and-global-development-76167

26 IDA. IDA18 replenishment final meeting—donor and borrower statements [Internet]. Washington (DC): World Bank; c 2017 [cited 2017 Nov 1]. Available from: https://ida.worldbank.org/content/ida18-replenishment-final-meeting-donor-and-borrower-statements

29 Government of Canada. Prime Minister hosts successful Global Fund Replenishment Conference to fight AIDS, Tuberculosis, and Malaria [Internet]. Montreal: Government of Canada; 2016 Sep 17 [cited 2017 Oct 25]. Available from: http://pm.gc.ca/eng/news/2016/09/17/prime-minister-hosts-successful-global-fund-replenishment-conference-fight-aids

30 Dreher A, Fuchs A. Rogue aid? An empirical analysis of China’s aid allocation. Can J Econ. 2015;48(3): 988–1023.

31 New Development Bank. About us [Internet]. Shanghai: NDB; [cited 2017 Nov 2]. Available from: http://www.ndb.int/about-us/organisation/members/

